# Exploring the role of LOX family in glioma progression and immune modulation

**DOI:** 10.3389/fimmu.2025.1512186

**Published:** 2025-04-09

**Authors:** Chen Liu, Huilian Qiao, Hongqi Li, Xiaolong Hu, Maohui Yan, Zhiguang Fu, Hengheng Zhang, Yingjie Wang, Nan Du

**Affiliations:** ^1^ Medical School of Chinese People’s Liberation Army (PLA), Beijing, China; ^2^ Department of Oncology, the Fifth Medical Center, Chinese People’s Liberation Army (PLA) General Hospital, Beijing, China; ^3^ Department of Radiotherapy, Air Force Medical Center, The Fourth Military Medical University, People’s Liberation Army (PLA), Beijing, China; ^4^ Department of Pathology, Air Force Medical Center, The Fourth Military Medical University, People’s Liberation Army (PLA), Beijing, China; ^5^ Department of Radiation Oncology, Beijing Geriatric Hospital, Beijing, China

**Keywords:** glioma, LOX family, immune infiltration, prognostic biomarker, therapeutic target, immune checkpoint inhibitor

## Abstract

**Background:**

Glioma is a major cause of mortality among central nervous system tumors, with a generally poor prognosis. The lysyl oxidase (LOX) family, a group of copper-dependent amine oxidases, has been implicated in the progression of various cancers, but its specific role in glioma and its relationship with immune infiltration remains insufficiently explored. This study aims to investigate the LOX family’s expression, prognostic significance, and immune infiltration dynamics in glioma to identify potential therapeutic targets.

**Methods:**

A comprehensive analysis was conducted using public databases to assess gene expression, mutation frequency, and immune infiltration patterns related to the LOX family in glioma. The results were validated through survival analysis and immunohistochemistry. Functional assays, including EdU, Transwell, and flow cytometry, were used to evaluate glioma cell proliferation, migration, invasion, and apoptosis. Co-culture experiments with immune cells, ELISA, and a glioma transplantation model were employed to study the immune-modulatory effects of the LOX family. Gene and protein expression levels were further analyzed using qRT-PCR and Western blotting.

**Results:**

The LOX family was significantly upregulated in low-grade gliomas and strongly associated with poor clinical outcomes. Although mutation frequencies were low, the LOX family contributed to glioma progression through pathways involving metastasis, hypoxia response, angiogenesis, and immune cell infiltration. LOX expression correlated with increased infiltration of macrophages and eosinophils and decreased presence of Treg and CD8+ T cells. Knockdown of LOX genes impaired glioma cell functions, induced apoptosis, and altered immune cell behavior by reducing M2 macrophage polarization and enhancing CD8+ T cell activity.

**Conclusions:**

The LOX family is overexpressed in glioma and is associated with poor prognosis and altered immune infiltration patterns. These findings highlight the LOX family as a promising prognostic marker and therapeutic target, particularly for enhancing the effectiveness of immunotherapy in glioma treatment.

## Introduction

Glioma is a prevalent malignant tumor in the central nervous system, and it is typically associated with a poor prognosis (1–3). Despite the significant advances in cancer treatment in recent years, the five-year survival rate for gliomas remains alarmingly low (4–6). Currently, the primary treatment methods include surgery, radiation therapy, and chemotherapy. However, their effectiveness is limited and often accompanied by significant side effects (7–10). As a result, identifying effective therapeutic targets and biomarkers has emerged as a crucial focus in glioma research (11–13). Gaining insight into the molecular mechanisms of glioma and its related biomarkers facilitates early diagnosis and establishes the foundation for personalized treatment (6, 14, 15).

The LOXs family, which consists of extracellular copper-dependent amine oxidase enzymes, is observed to be upregulated in numerous types of cancers, such as breast cancer, lung cancer, and colorectal cancer (16–19). The LOXs family promotes tumor invasion and metastasis (20, 21). These proteins are involved in diverse biological processes, including cell migration, remodeling of the extracellular matrix, and generation of blood vessels, thereby exerting an influence on tumor growth and metastasis (22, 23). However, the existing research regarding the role and significance of the LOXs family in glioblastoma is currently limited (19).

Research on the LOX family in gliomas has attracted increasing attention recently. Studies have shown that the expression levels of LOX are significantly higher in glioma tissues compared to normal brain tissue and are positively correlated with the malignancy of the tumor (24). LOX enhances the invasive ability of glioma cells by promoting the crosslinking and remodeling of the extracellular matrix (25). Furthermore, the expression of LOX family members LOXL1 and LOXL2 in gliomas is also associated with tumor progression and poor prognosis (24). However, the specific mechanisms of LOX family involvement in gliomas, particularly its role in immune infiltration, remain controversial.

Immune infiltration plays a significant role in the tumor microenvironment (26, 27). Different types of immune cells, such as macrophages (28–30). T cells (31–33) With B cells (34–36) The growth and metastasis of tumors may be influenced by various mechanisms. Immune infiltration in gliomas is considered to be closely associated with tumor prognosis and treatment response (37–39). However, the impact of the LOXs family on immune infiltration in gliomas remains uncertain.

Despite the substantial research on the LOX family and immune infiltrates in various cancers, the interactions and effects of these factors in gliomas have not received sufficient attention (40, 41). This research gap hampers our comprehensive understanding of the intricate biological characteristics of gliomas and impedes the advancement of novel treatment strategies.

Based on the background mentioned above, this study aims to thoroughly examine the expression patterns of the LOXs family in gliomas, evaluate its prognostic value, and explore its relationship with immune infiltration. We utilized multiple publicly available databases and performed a comprehensive analysis using bioinformatics and laboratory techniques. This study is valuable because it has the potential to not only identify LOXs family as novel biomarkers for glioma prognosis but also to offer new targets and strategies for personalized treatment of glioma.

## Materials and methods

### Analysis of the expressed levels of the LOX family

We utilized the web-based platform GSCA (http://bioinfo.life.hust.EdU.cn/GSCA/#/), which integrates genomics and immunogenomics, to study the expression levels of LOX in 33 different cancer types. Our objective was to investigate cancer gene sets. The expression of LOX family members in tumors and normal tissues was investigated using UALCAN (http://ualcan.path.uab.EdU/index.html), an interactive web portal designed for the in-depth analysis of cancer genomic profiles based on The Cancer Genome Atlas (TCGA) gene expression data. Furthermore, we investigated the expression of the LOX family in glioma patients with varying races, ages, tumor grades, and other clinical-pathological features by employing UALCAN (42–45).

### LOX family gene variation and correlation analysis

In our analysis of LOX family variants in TCGA glioblastoma samples, we utilized the cBio Cancer Genomics Portal (http://cbioportal.org), an open-access resource that allows interactive exploration of multidimensional cancer genomics datasets. Furthermore, it is also utilized to evaluate the correlation among members of the LOX family (46–48).

### Gene-gene interaction and protein-protein interaction network

GeneMANIA (http://genemania.org/) is a tool for efficiently constructing gene networks and predicting their functions within the context of conserved genes. Conversely, STRING (https://string-preview.org/) is a functional enrichment analysis tool specifically designed for protein-protein interaction networks. Both tools investigate LOX family genes and protein networks (45, 49, 50).

### Survival analysis

The Kaplan-Meier plotter, available at www.kmplot.com, is an online database containing gene expression and clinical data. LinkedOmics, accessible at http://www.linkedomics.org/login.php, is a public portal website that provides multi-omics data for 32 types of TCGA cancer. UALCAN and TISIDB are tools commonly employed to study the correlation between LOX family expression and survival (51–53).

### Enrichment analysis of function

We utilized CancerSEA, the first specialized database designed to thoroughly interpret cancer cell functional states at the single-cell level, to investigate the functionality of the LOX family expression (54). Gene lists can be transformed into a biological perspective for pathway analysis of LOXs using the WEB-based Gene Set Analysis Toolkit (WebGestalt) (55–57).

### Immunosuppressant analysis

TISIDB is a web portal focusing on the interactions between tumors and the immune system. It combines various data types to investigate the correlation between LOX family members and immune inhibitors (58, 59).

### Analysis of tumor-infiltrating immune cells

We first utilize the ssGSEA algorithm from the R package “GSVA” to visualize the enrichment levels of 24 common immune cell types (60), which include plasma-like DC(pDC), CD8^+^ T cells, dendritic cells (DC), T gamma delta (Tgd), T central memory (Tcm), regulatory T cells (Treg), T helper cells (Th), T effector memory (Tem), NK 56bright cells, NK 56dim cells, T follicular helper cells (TFH), mast cells, Th1 cells, Th17 cells, immature DC (iDC), B cells, cytotoxic cells, Th2 cells, natural killer (NK) cells, activated DC (aDC), neutrophils, T cells, macrophages, and eosinophils.

The relationship between LOX expression and immune cell infiltration was evaluated using Spearman correlation analysis. Furthermore, the levels of immune cell infiltration were compared between the high LOXs expression group and low LOXs expression group using the Mann-Whitney U test (61–63).

### Cell culture and manipulation

The human astrocyte cell line Heb (YS003C, Shanghai Yaji Biological Technology Co., Ltd., http://www.yajimall.com/), the human glioblastoma cell line T98G (CBP60301, Nanjing CoBioer Biological Technology Co., Ltd., https://www.cobioer.com/), LN-229 (CBP60302, Nanjing CoBioer Biological Technology Co., Ltd.), and the mouse glioma cell line GL261 (CBP60669, Nanjing CoBioer Biological Technology Co., Ltd.) were cultured in DMEM medium (11960044, Gibco, USA, https://www.thermofisher.cn/cn/zh/home.html) supplemented with 10% fetal bovine serum (10099141C, Gibco, USA) and 1% penicillin/streptomycin (15140122, Gibco, USA).

T98G and GL261 cells were collected during the logarithmic growth phase and then infected with lentivirus to silence the LOX family factors LOX, LOXL1, LOXL2, LOXL3, and LOXL4. The cell groups included the sh-NC group (infected with sh-NC lentivirus), sh-LOX-1 group (infected with sh-LOX-1 lentivirus), sh-LOX-2 group (infected with sh-LOXL-2 lentivirus), sh-LOXL1-1 group (infected with sh-LOXL1-1 lentivirus), sh-LOXL1-2 group (infected with sh-LOXL1-2 lentivirus), sh-LOXL2-1 group (infected with sh-LOXL2-1 lentivirus), sh-LOXL2-2 group (infected with sh-LOXL2-2 lentivirus), sh-LOXL3-1 group (infected with sh-LOXL3-1 lentivirus), sh-LOXL3-2 group (infected with sh-LOXL3-2 lentivirus), sh-LOXL4-1 group (infected with sh-LOXL4-1 lentivirus), sh-LOXL4-2 group (infected with sh-LOXL4-2 lentivirus). The specific sequences are listed in **Supplementary Table S1**. The lentivirus used in this study was constructed and provided by Shanghai Sangon Biotech Co., Ltd. (https://www.sangon.com/). The following procedure was used for infection: Logarithmic growth phase T98G and GL261 cells were collected, and 5×104 cells/mL cell suspension was prepared. The suspension was then seeded in a 6-well plate with 2 mL per well. The plate was incubated overnight at 37°C. Subsequently, each well was infected with a recombinant lentivirus at a final concentration of 1×10^8^ TU/mL. After 24 hours of infection, the cells were treated with puromycin (2 µg/mL, 540222, Sigma-Aldrich, https://www.sigmaaldrich.cn/CN/zh) for 7 days to establish stable infected cell lines for further experiments (64, 65).

### qRT-PCR

Total RNA was extracted from the cells using TRIzol (Invitrogen, USA) (15596026). The concentration and purity of the extracted total RNA were measured using a Nanodrop 2000 micro UV spectrophotometer (Nanodrop, USA) (1011U). The reverse transcription of mRNA into cDNA followed the instructions from the PrimeScript RT reagent Kit (RR047A, Takara, Japan, https://www.takarabiomed.com.cn/). The ABI7500 Quantitative PCR Instrument (7500, ABI, USA) should be used for real-time fluorescence quantitative PCR detection. The reaction conditions are as follows: pre-denaturation at 95°C for 10 min, denaturation at 95°C for 10 s, annealing at 60°C for 20 s, and extension at 72°C for 34 s, for a total of 40 cycles. Using GAPDH as an internal control/reference. The relative transcription level of the target gene is determined using the comparative quantification method known as the 2^-△△CT^ method. △△Ct is calculated by subtracting △the control group’s Ct front of the \textit △Ct represents the difference in Ct values between the target gene and the reference gene. The relative transcription level of the target gene is then calculated as 2^-△△^Ct (66). Each experiment was replicated three times, and the primers used were synthesized by the TaKaRa Company (**Supplementary Table S2**).

### Western blot

Total protein was extracted from cells using RIPA lysis buffer containing PMSF (P0013B, Beyotime, Shanghai, https://www.beyotime.com/index.htm), and quantitative analysis was conducted with the BCA Protein Assay Kit (23225, Thermo Fisher Scientific, USA). The protein (50 μg) was dissolved in 2x SDS loading buffer and boiled at 100°C for 5 min. Each sample was then subjected to SDS-PAGE gel electrophoresis. The proteins were transferred onto a polyvinylidene fluoride (PVDF) membrane using the wet transfer method. The membrane was blocked with 5% skim milk at room temperature for 1 h. Subsequently, the PVDF membrane was incubated with a diluted primary antibody (see **Supplementary Table S3** for detailed information) overnight at 4°C. The membrane was washed thrice with Tris-buffered saline containing 0.1% Tween-20 (TBST) for 10 min each time. Then, the membrane was incubated with an HRP-conjugated goat anti-rabbit IgG H&L secondary antibody (ab97051, 1:2000, Abcam, UK) for 1 h. After additional washing with TBST, the membrane was finally placed on a clean glass plate. Take the appropriate amounts of Solution A and B from the Pierce™ ECL Western Blot Substrate Kit (32209, Thermo Fisher Scientific, USA). Thoroughly mix the solutions in a darkroom and apply them to the membrane. Lastly, capture an image using the Bio-Rad imaging system (ChemiDoc™ XRS+, BIO-RAD, USA) (66).

### EdU experiment

Inokuluj komórki do 24-dołkowych płyt, wykonując trzy replikacje dla każdej grupy komórek. EdU (C10310-1, Guangzhou Ribobio Biotechnology Co., Ltd., https://www.ribobio.com/) was added to the culture medium at a final concentration of 10 µmol/L, followed by incubation in a culture chamber for 2 hours. The culture medium was removed, followed by fixation in a PBS solution containing 4% paraformaldehyde at room temperature for 15 minutes. Afterwards, the samples were washed twice with a PBS solution containing 3% BSA. The samples were incubated at room temperature with 0.5% Triton-100 (HFH10, Invitrogen™, USA) in PBS for 20 minutes. After incubation, the samples were washed twice with PBS containing 3% BSA. Add 100 µl of EdU staining solution to each well. Incubate at room temperature in the dark for 30 minutes. Dodaj DAPI (C1002, Beyotime, Shanghai) do zabarwienia jądra na 5 minut, a następnie losowo obserwuj 6-10 pól pod mikroskopem fluorescencyjnym po zalaniu preparatów, rejestrując liczbę komórek dodatnich w każdym polu (67). The EdU labeling rate was calculated as the percentage of positive cells over the sum of positive and negative cells, multiplied by 100%. This calculation was repeated three times in each experiment.

### Detection of cell migration and invasion using Transwell

Extracellular migration and invasion experiments were performed using a Transwell chamber (3422, Corning, USA) with 8 µm pore size in a 24-well plate. The Matrigel gel (354277, BD Biosciences, USA) was retrieved and thawed to a liquid state for the invasion experiment. The gel had been stored at -80°C by the manufacturer’s instructions and was equilibrated overnight at 4°C. Dilute the matrix gel by adding 200 µl Matrigel to 200 µl serum-free culture medium, ensuring thorough mixing. Then, add 50 µl of the diluted mixture to each well of the Transwell plate. Place the plate in a cell incubator for 2-3 hours until the gel solidifies. Add 200 μL of cell suspension to each well in the upper chamber and 800 μL of medium containing 20% fetal bovine serum (FBS) to the lower chamber. After incubating at 37°C for 24 hours, the Transwell chambers were removed, and the inner layer of cells on the Transwell membrane was wiped using a cotton swab. The cells were then rinsed twice with PBS and fixed in 4% formaldehyde within the chamber. Afterward, they went through three washes with water before being incubated with 0.1% crystal violet (C0121, Beyotime, Shanghai) for 30 minutes (68). Cells in five areas were captured and counted using the Nikon Eclipse Ci optical microscope (Nikon, Tokyo, Japan). The counting was repeated for each sample to quantify the cells.

### Glioma cells were co-cultured with either macrophages or CD8^+^ T cells

M2 polarization was induced in THP-1 cells by seeding 1×10^6^ cells (CBP60518, Nanjing KeyGen Biotech Co., Ltd.) in a 6-well plate and culturing them at 37°C, 5% CO2. Phorbol 12-myristate 13-acetate (PMA, 100 ng/mL, P8139, Sigma-Aldrich, USA) was added to induce THP-1 cell differentiation into macrophages for approximately 12 hours. After 12 hours, most cells will adhere to the cell wall. Next, the culture medium should be changed, and IL-4 (20 ng/mL, P5129, Beyotime, Shanghai) and IL-13 (20 ng/mL, P5178, Beyotime, Shanghai) should be added to induce M2 macrophage polarization for approximately 24 hours.

In the coculture experiment, glioma cells were co-cultivated with either macrophages or CD8+ T cells. Specifically, 2 × 10^5^ glioma cells were added to the upper chamber of Transwell wells. Place glioma cells and either macrophages or CD8^+^ T cells into the lower chamber of the Transwell insert. Joint cultivation for 24 hours. Following the completion of the experiment, various methods can be employed to analyze glioma cells, macrophages, and CD8^+^ T cells. The macrophages or CD8^+^ T cells used in this study were obtained from QINGQI (Shanghai) Biotechnology Development Co., Ltd. (BFN60810741) (69).

### Flow cytometry

Cell apoptosis rate was assessed by collecting glioma cells or macrophages after 24 hours of co-culturing in a flow tube. The collected cells were then centrifuged, and the supernatant was discarded. Wash the cells three times using cold PBS and remove the supernatant after centrifugation. To prepare the Annexin-V-FITC/PI staining solution, follow the instructions provided in the Annexin-V-FITC Cell Apoptosis Detection Kit (C1062, Beyotime, Shanghai), which involves mixing Annexin-V-FITC, PI, and HEPES buffer in a ratio of 1:2:50. Resuspend 1×10^6^ cells in 100 μL of staining solution. Mix by shaking and incubate at room temperature for 15 minutes. Afterward, add 1 mL of HEPES buffer and mix again by shaking (70). Cellular apoptosis was assessed by detecting FITC and PI fluorescence at 525 nm and 620 nm, respectively. The experiment was performed three times.

Cell cycle: Harvest the cells and wash them three times with PBS. Fix the cells overnight in 75% pre-chilled ethanol. Before experimentation, pre-treat the cells with PI and RNaseA according to the guidelines outlined in the Cell Cycle Detection Kit (C1052, Beyotime, Shanghai). The flow cytometer (C500, Beckman, USA) was utilized to detect and analyze the cell cycle at various stages, employing FlowJo software (71).

Detection of the M2 macrophage ratio: Mouse tumor tissues were collected in cold PBS and processed following the provided description. The samples were mechanically sliced using the McIlwain Tissue Chopper (Mickle Laboratory Engineering). Subsequently, the samples were incubated in serum-free medium at 37°C in a shaking water bath for 1 hour, along with 3 mg/ml Collagenase Type A (10103578001, Roche, USA) and 25 μg/ml DNase I (11284932001, Roche, USA). Harvest the macrophages induced by co-culturing THP-1 cells by using cold PBS. The cells were resuspended in flow cytometry buffer, specifically 1x PBS buffer containing 1% BSA. Then, they were stained with an anti-CD163 antibody (333618, 1:100, Biolegend, USA) and an anti-CD206 antibody (321110, 1:100, Biolegend, USA) while keeping them on ice for 30 minutes. After that, the stained cells were analyzed using a flow cytometer (C500, Beckman, USA) and the data was further analyzed using FlowJo software (69).

### Construction of subcutaneous tumor transplantation in mice

All animal experiments conducted in this study have received approval from our institution’s Animal Ethics Committee and strictly adhere to internationally recognized guidelines for animal welfare and the principles of replacement, reduction, and refinement (3R). Experimental animals are provided with appropriate nutrition and care to minimize their level of distress. Following the completion of the experiment, all animals will be euthanized using a humane method.

Forty-eight healthy male clean-grade C57BL/6 mice, aged 6-8 weeks, weighing 20-25 g, were obtained from Beijing Vital River Laboratory Animal Technology Co., Ltd (219, http://m.vitalriver.com/home). Mice breeding and animal experiments are conducted in our experimental animal center in adherence to internationally recognized animal welfare and ethics standards. The mouse groups included sh-NC, sh-LOX, sh-LOXL1, sh-LOXL2, sh-LOXL3, and sh-LOXL4. Stable silenced GL261 cells were injected subcutaneously into the right dorsal area of C57BL/6 mice using lentivirus at a concentration of 1 × 10^7^ cells/100 µl. Each mouse received a subcutaneous injection of tumor cells on the right side, with 5 × 10^6^ fused cells injected per mouse. The health condition and behavior of mice were monitored daily. Tumor volume and mass were calculated by collecting tumor tissues from mice euthanized using the cervical dislocation method 21 days after inoculating tumor cells. The formula for tumor volume calculation is as follows: 0.5*a*b^2^ (where a represents the long axis and b represents the short axis). A caliper was used to measure the length and width of the tumor (72).

Subcutaneous injection was performed using U87MG glioblastoma cells (labeled with GFP; ATCC, USA). Each mouse was inoculated subcutaneously with 1×10^6^ cells. The cells were suspended in 100 μL of PBS buffer (Gibco, USA) (73–75).

### Immunofluorescent staining

Organ slices were fixed in 4% formaldehyde (P0099, Beyotime, Shanghai), permeabilized with 0.3% Triton X-100 (P0096, Beyotime, Shanghai), and blocked with 1% bovine serum albumin (ST023, Beyotime, Shanghai). The sections were then incubated overnight at 4°C with primary antibodies Ki67 (1:500, ab279653; Abcam, UK) and CD8 (1:800, ab228965; Abcam, UK). Following PBS washing, the sections were subsequently incubated with secondary antibodies. These included sheep anti-mouse Alexa Fluor^®^ 647-conjugated secondary antibody (1:2000, ab150115, Abcam, UK) or sheep anti-rabbit Alexa Fluor^®^ 488-conjugated secondary antibody (1:2000, ab150077, Abcam, UK). Finally, we captured images using a FluoView FV10i confocal laser scanning microscope equipped with a 40x objective lens (Olympus, Japan). Five random slices were selected for each mouse, and each slice was photographed from three different fields of view. Subsequently, we calculated the percentages of KI67-positive cells and CD8+ T cells (76).

### Detection of immune factor expression by ELISA

To obtain the culture supernatant from co-culturing glioma and CD8^+^ T cells, the preserved mouse sera from each group were collected and then centrifuged at 1500 g for 15 minutes. Following the instructions provided for ELISA antibody usage, INF-γ (human: E-EL-H0108c, Elabscience, Wuhan; mouse: E-EL-M0048c, Elabscience) and TNF-α (human: E-EL-H0109c, Elabscience; mouse: E-EL-M3063, Elabscience) ELISA kits were used to accurately measure the levels of immune factors INF-γ and TNF-α by strictly following the operational steps. 100 µl of dilution buffer, 100 µl of the specimen, and 100 µl of standard were added to the well. The mixture was then incubated at 37°C for 90 minutes before removal. Next, add 100 µl of biotinylated detection antibody to the well and incubate at 37°C for one hour. Proceed to wash the samples three times, each for two minutes. Next, add 100 µl of the horseradish peroxidase conjugate to the well and incubate it at 37°C for 30 minutes. After washing five times, add the substrate reagent and incubate in the dark at 37°C for 15 minutes. Then, stop the reaction. The absorbance at 450 nm was measured using the Epoch microplate spectrophotometer (Bio-Tek, Winooski, VT, USA) (77). Each sample is set with three multiple holes.

### Statistical analysis

All data is tested for normality first. Continuous variables are represented as the mean ± standard deviation (SD). Use unpaired t-test for comparison between two groups. One-way analysis of variance (ANOVA) is used for comparisons involving three or more groups. Multiple comparison corrections, such as Tukey or Bonferroni, will be applied if necessary. Data deviating from a normal distribution will be analyzed using either the Mann-Whitney U or Kruskal-Wallis tests. Most of our bioinformatics analyses are conducted using the R software, while the rest are performed with SPSS and GraphPad Prism 6.0. Single-factor and multi-factor analyses are utilized to evaluate the influence of clinical variables on survival. A p-value less than 0.05 is considered statistically significant (78–80).

## Results

### Diverse expression of the LOX family in different types of cancer: using glioma as a specific example

**Figure 1:**
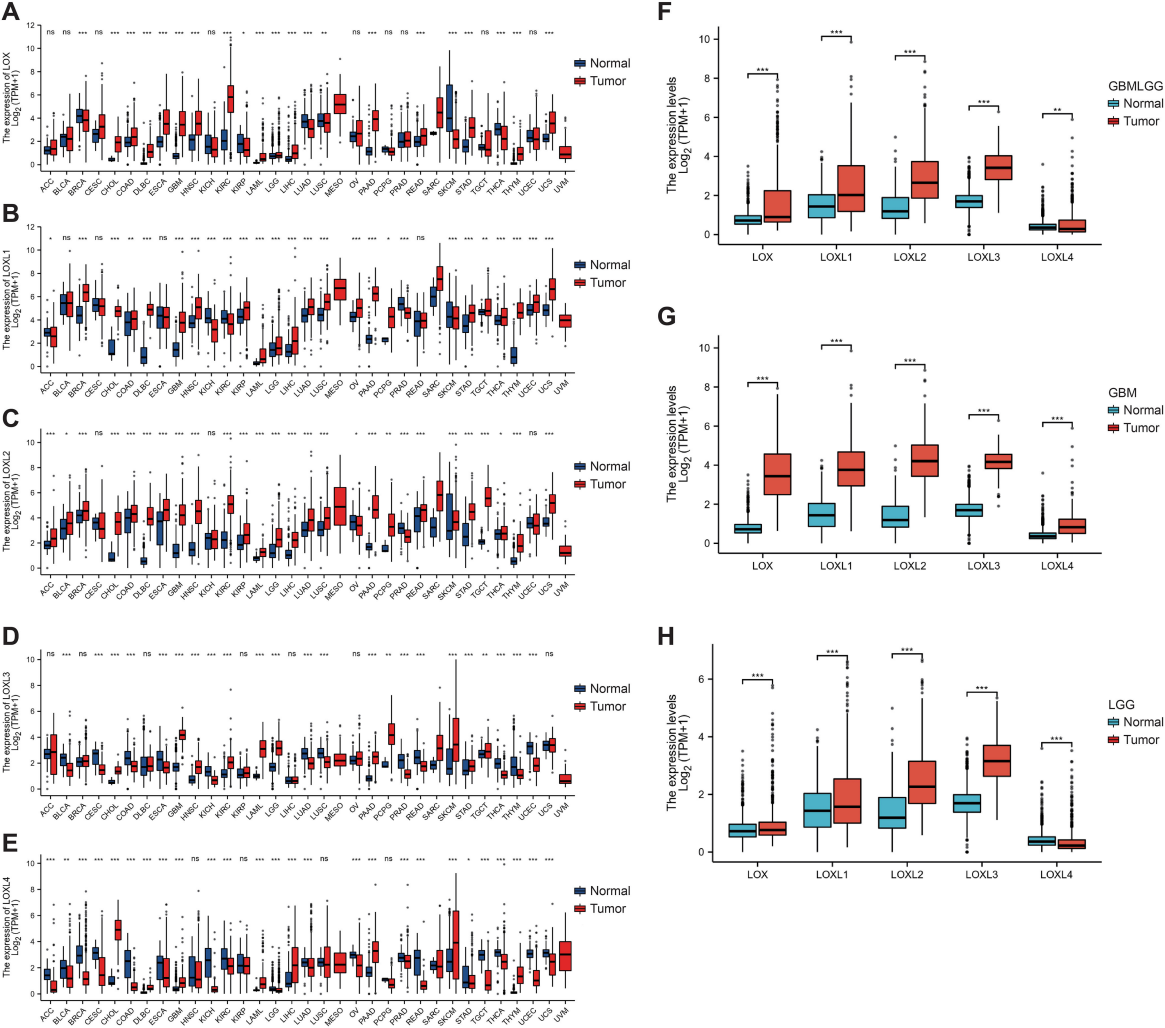
Expression of the LOX family in glioma and normal tissue. The GSCA method was used to examine the expression levels of the LOX family in different tumor types from the TCGA database. **(A–E)** Expression levels of the LOX family in different tumor types. **(F–H)** Expression of the LOX family in glioma is significantly higher than in normal tissues, as shown by UALCAN and RFEET. *P < 0.05, **P < 0.01, ***P < 0.001; ns, not significant.

To examine variations in LOX expression between tumors and normal tissues across various cancer types, we employed GSCA to analyze LOX mRNA levels. The results revealed a significant increase in mRNA levels of the LOX family in 13 types of cancer, including GBM (glioblastoma, WHO grade IV) and LGG (low-grade glioma, WHO grade II-III), which indicates the potential pivotal role of the LOX family in neurogliomas (**Figure 1A**). **Figures 1B-E** further demonstrate the high expression of LOXL1, LOXL2, LOXL3, and LOXL4 in GBM and LGG, consistent with the results presented in **Figure 1A**. It provides additional evidence supporting the widespread upregulation of the LOX family in these two types of cancer. Furthermore, we utilized UALCAN technology to analyze the differential expression of LOX family members in both glioma tumors and non-tumor tissues. The TCGA dataset was used for validation, and the results demonstrated that the expression of the LOX family was significantly higher in glioblastoma (GBM) and low-grade glioma (LGG) compared to normal tissues. Validation using the TCGA dataset showed that the expression of the LOX family is significantly higher in both GBM (WHO grade IV) and LGG (WHO grade II-III) compared to normal tissues (**Figures 1F-H**). These results indicate that the high expression of the LOX family in gliomas exhibits significant heterogeneity, supporting the hypothesis that the LOX family may play an important role in the initiation and progression of gliomas.

### The robustness of the LOX family genome and its role in signaling networks

**Figure 2:**
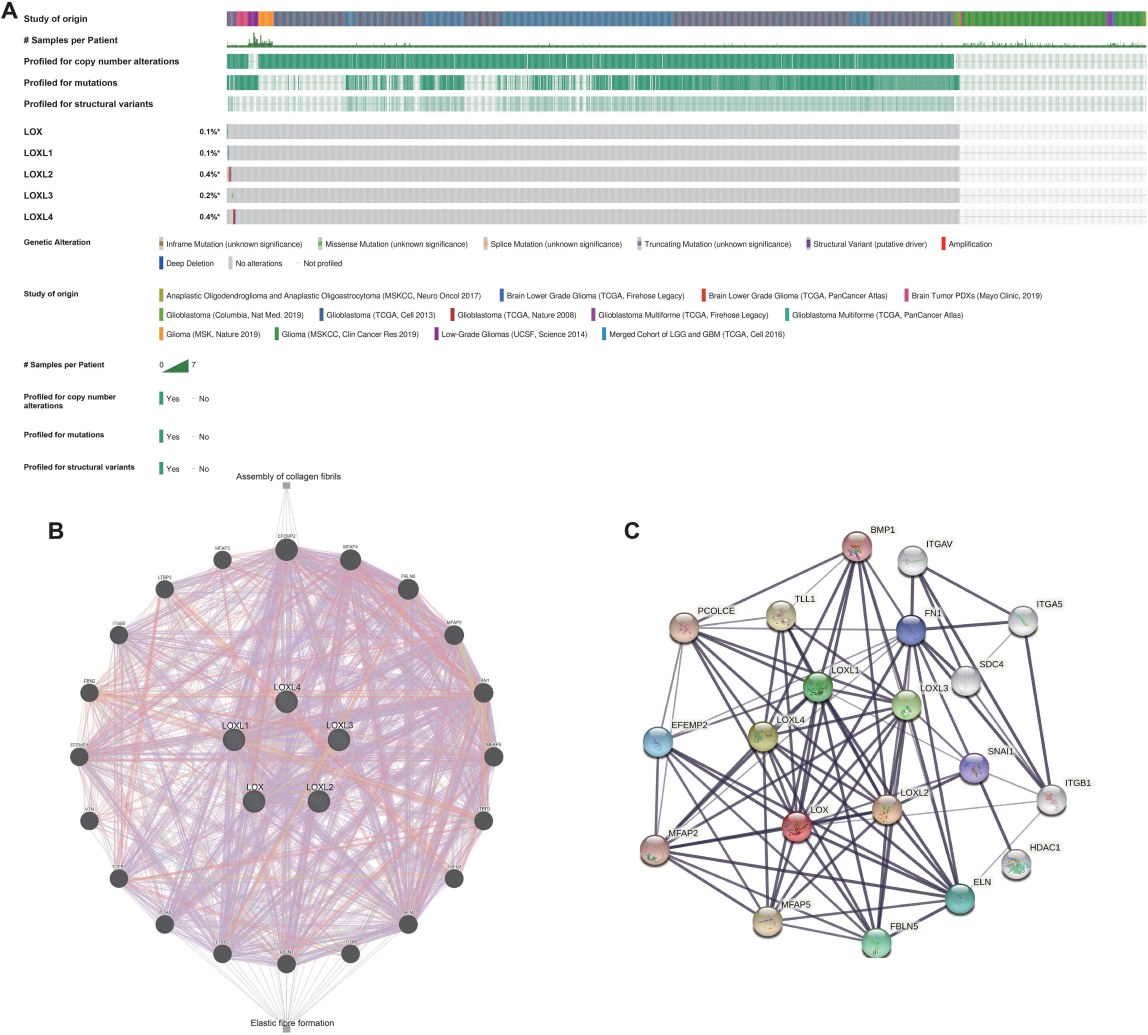
Genomic alterations of LOX family members and gene-gene/protein-protein interaction networks of LOX family genes. **(A)** This paper summarizes the genomic alterations of the LOX family in TCGA glioma samples. **(B)** The gene network associated with the LOX family genes was constructed using GeneMANIA. **(C)** A protein-protein interaction network involving the LOX family genes was generated using STRING.

Subsequently, bioinformatics is employed to identify the types and frequencies of alterations in the LOX family in TCGA glioma samples. The results reveal that the mutation rate of LOX family members is below 0.5%. This data implies the genomic stability of the LOX family in glioma samples, as depicted in **Figure 2A**. Next, we conducted a network analysis of gene-gene and protein-protein interactions, which uncovered 20 potential target genes and 15 potential proteins related to the LOX family. These findings offer valuable insights for future investigations into the involvement of the LOX family in glioblastoma (**Figures 2B, C**). The low mutation rate of the LOX family in gliomas, along with its extensive network of signaling interactions, suggests that it may influence tumor biological behavior through non-mutational mechanisms, such as the regulation of gene expression.

### Expression of LOX family and its correlation with prognosis in glioblastoma

**Figure 3:**
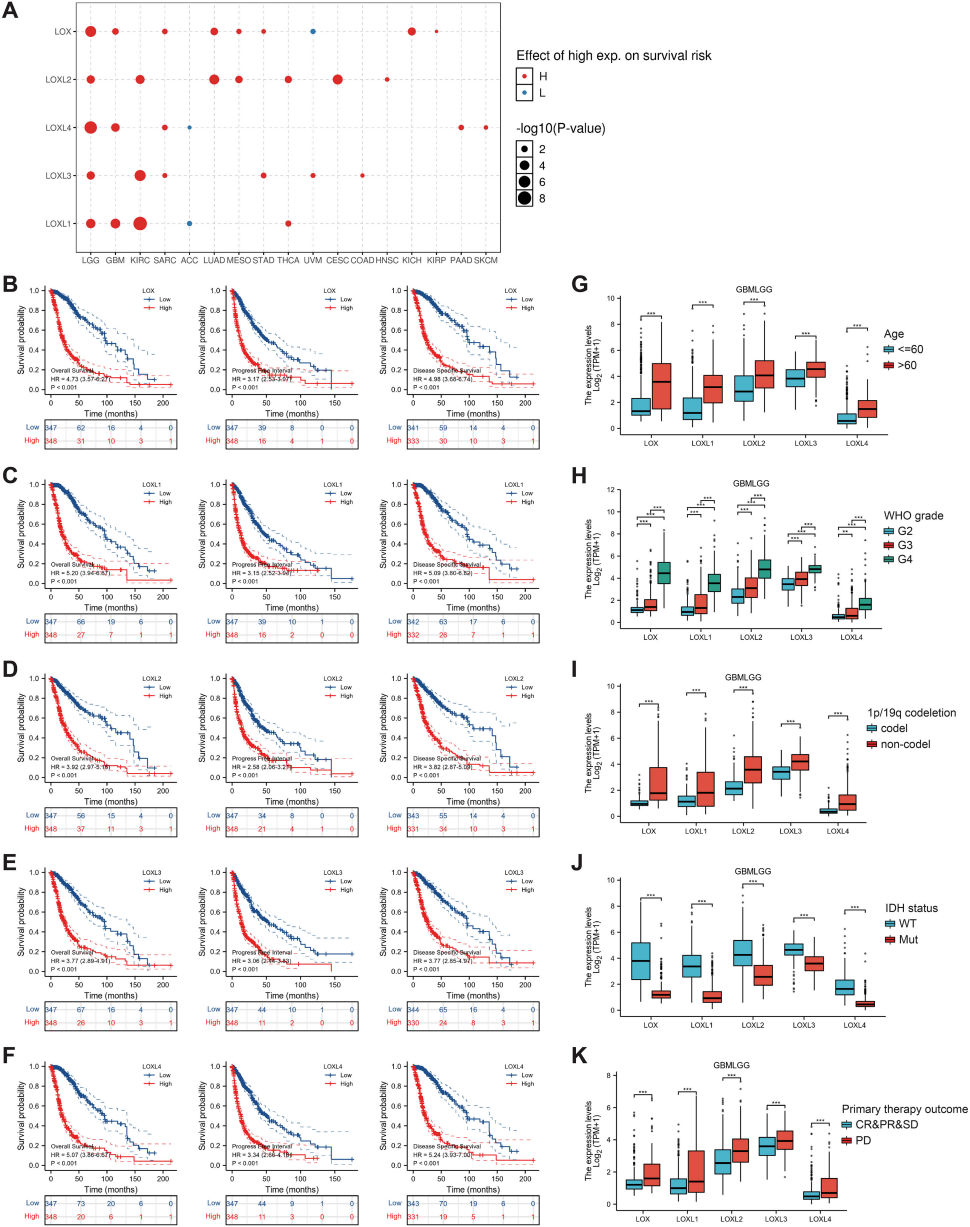
Prognostic value of LOX family members in glioma. **(A)** Survival risks between high and low expression of LOX family genes across 33 cancer types from TCGA. **(B–F)** Survival curves for individual LOX family members were analyzed using R language. **(G-K)** Clinical and pathological glioma features with different LOX family expression levels. *P < 0.05, **P < 0.01, ***P < 0.001.

Subsequently, we examined the correlation between the LOX family members’ expression levels and the glioma prognosis. We utilized the GSCALite tool to establish a noteworthy association between elevated expression of the LOX family and the risk of survival in various gliomas, as shown in **Figure 3A**. We analyzed the prognostic data related to the LOX family using the R language based on the study published in ‘Cell’ by Liu J. The results revealed a strong association between high expression and unfavorable prognosis, including overall survival (OS), progression-free survival (PFS), and disease-specific survival (DSS) (**Figures 3B-F**). To investigate LOX’s impact on glioblastoma’s clinical features, we analyzed the clinical and pathological characteristics of glioblastoma cases with varying expressions of the LOX family. **Figures 3G-K** and **Table 1** demonstrate that the LOX family’s high expression correlates with various clinical and pathological features such as age and WHO grade. It provides robust evidence supporting the involvement of the LOX family in glioma prognosis. Subsequently, we conducted univariate and multivariate Cox regression analyses to further investigate the prognostic significance of the LOX family in gliomas. In the univariate Cox regression analysis, high expression of LOX/LOXL1/LOXL3/LOXL4 was identified as an independent risk factor for overall survival (hazard ratio [HR] = 4.733, 5.256, 3.922, 3.784, 5.151, P < 0.001). Moreover, factors such as age, WHO grading, 1p/19q co-deletion, IDH status, and other clinical characteristics have also shown predictive benefits for clinical outcomes in univariate Cox regression analysis. In multivariate Cox regression analysis, high expression of LOXL4 (HR: 2.095, P < 0.001), age > 60, WHO grade G3 G4, and wild-type IDH were the only statistically significant factors (**Table 2**). These results confirm the close relationship between LOX family expression and glioma prognosis, supporting the hypothesis that the LOX family could be a potential prognostic biomarker for gliomas.

**Table 1:** Clinicopathological characteristics of glioma with different LOX family expression.

Characteristic	Low expression of LOX	High expression of LOX	p	Low expression of LOXL1	High expression of LOXL1	p	Low expression of LOXL2	High expression of LOXL2	p	Low expression of LOXL3	High expression of LOXL3	p	Low expression of LOXL4	High expression of LOXL4	p
n	348	348		348	348		348	348		348	348		348	348	
**WHO grade, n (%)**			<0.001			<0.001			<0.001			<0.001			<0.001
																G2	175 (27.6%)	49 (7.7%)		169 (26.6%)	55 (8.7%)		172 (27.1%)	52 (8.2%)		168 (26.5%)	56 (8.8%)		161 (25.4%)	63 (9.9%)	
G3	131 (20.6%)	112 (17.6%)		128 (20.2%)	115 (18.1%)		123 (19.4%)	120 (18.9%)		128 (20.2%)	115 (18.1%)		132 (20.8%)	111 (17.5%)	
G4	1 (0.2%)	167 (26.3%)		5 (0.8%)	163 (25.7%)		10 (1.6%)	158 (24.9%)		13 (2%)	155 (24.4%)		14 (2.2%)	154 (24.3%)	
**IDH status, n (%)**			<0.001			<0.001			<0.001			<0.001			<0.001
																WT	21 (3.1%)	225 (32.8%)		15 (2.2%)	231 (33.7%)		49 (7.1%)	197 (28.7%)		41 (6%)	205 (29.9%)		17 (2.5%)	229 (33.4%)	
Mut	325 (47.4%)	115 (16.8%)		330 (48.1%)	110 (16%)		296 (43.1%)	144 (21%)		302 (44%)	138 (20.1%)		327 (47.7%)	113 (16.5%)	
**1p/19q codeletion, n (%)**			<0.001			<0.001			<0.001			<0.001			<0.001
code	151 (21.9%)	20(2.9%)		115 (16.7%)	56 (8.1%)		144 (20.9%)	27 (3.9%)		136 (19.7%)	35(5.1%)		140 (20.3%)	31 (4.5%)	
non-codel	197 (28.6%)	321 (46.6%)		232 (33.7%)	286 (41.5%)		203 (29.5%)	315 (45.7%)		212 (30.8%)	306 (44.4%)		206 (29.9%)	312 (45.3%)	
**Age, meidan (IQR)**	39 (32, 50)	53 (40, 63)	<0.001	38 (31, 47)	55 (43, 63)	<0.001	40 (32, 52)	52 (37, 63)	<0.001	41 (33, 52)	52 (36, 63)	<0.001	39 (32,49)	54 (40,63)	<0.001

Bold values indicate statistically significant differences (P < 0.05).

**Table 2:** Univariate and multivariate analysis of the correlation of LOX expression with OS among glioma patients.

Characteristics	Total(N)	Univariate analysis		Multivariate analysis	
		Hazard ratio (95% CI)	P value	Hazard ratio (95% CI)	P value
Gender	695				
Male	398	Reference			
Female	297	0.793 (0.621-1.012)	0.062	0.803 (0.611-1.055)	0.115
Age	695				
<=60	552	Reference			
>60	143	4.668 (3.598-6.056)	**<0.001**	1.488 (1.094-2.025)	**0.011**
WHO grade	634				
G2	223	Reference			
G3	243	2.999 (2.007-4.480)	**<0.001**	1.838 (1.185-2.851)	**0.007**
G4	168	18.615 (12.460-27.812)	**<0.001**	4.326 (2.462-7.601)	**<0.001**
1p/19q codeletion	688				
codel	170	Reference			
non-codel	518	4.428 (2.885-6.799)	**<0.001**	1.214 (0.710-2.076)	0.479
IDH status	685				
Mut	439	Reference			
WT	246	8.551 (6.558-11.150)	**<0.001**	2.415 (1.446-4.033)	**<0.001**
LOX	695				
Low	348	Reference			
High	347	4.733 (3.570-6.275)	**<0.001**	1.230 (0.781-1.938)	0.371
LOXL1	695				
Low	348	Reference			
High	347	5.256 (3.980-6.942)	**<0.001**	1.028 (0.653-1.619)	0.906
LOXL2	695				
Low	348	Reference			
High	347	3.922 (2.978-5.166)	**<0.001**	0.938 (0.629-1.398)	0.754
LOXL3	695				
Low	348	Reference			
High	347	3.784 (2.905-4.928)	**<0.001**	1.034 (0.712-1.502)	0.859
LOXL4	695				
Low	348	Reference			
High	347	5.151 (3.920-6.768)	**<0.001**	2.095 (1.445-3.039)	**<0.001**

Bold values indicate statistically significant differences (P < 0.05).

### The LOX family and immune checkpoint inhibitors offer a new therapeutic perspective

**Figure 4:**
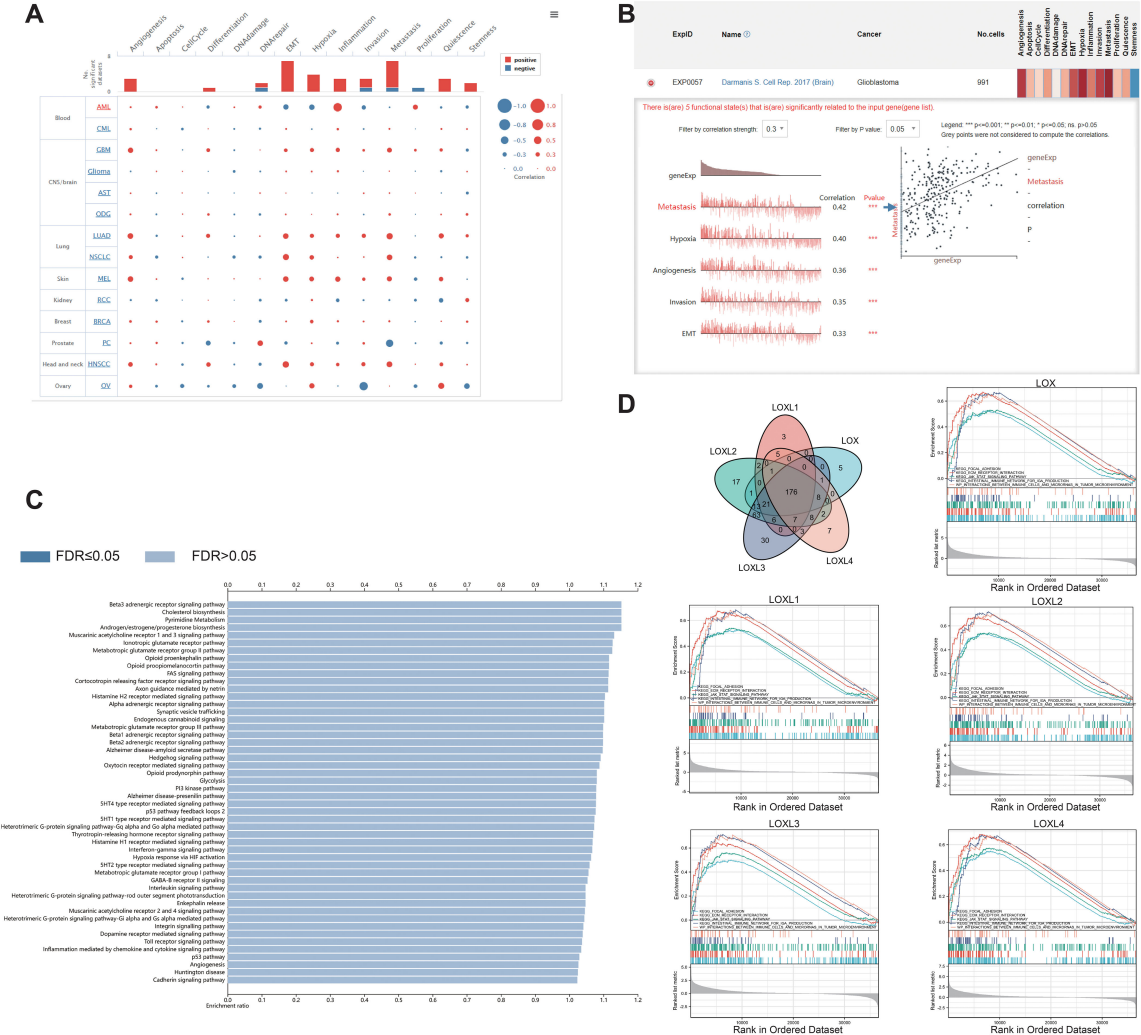
Functions of the LOX family in glioma. **(A)** Single-cell analysis revealed that LOXs primarily participate in metastasis, hypoxia, angiogenesis, invasion, and EMT. **(B)** CancerSEA data showed positive correlations between LOXs and metastasis, hypoxia, angiogenesis, invasion, and EMT. **(C)** WEGO analysis was conducted to explore the biological processes associated with LOXs. **(D)** A Venn diagram was employed to select 176 significantly enriched biological processes and pathways, with 5 visualized. ***P < 0.001.

To investigate the potential roles of LOX family members in glioblastoma, we performed single-cell analysis using CancerSEA. The findings revealed a positive association between LOX and multiple biological processes, including metastasis and hypoxia (**Figures 4A, B**). We utilized WebGestalt to conduct pathway analysis on LOX and found that LOX expression is linked to nervous system signal transmission, immune infiltration, and biosynthesis (**Figure 4C**). Moreover, we conducted a single-gene GSEA analysis and excluded 176 biological processes and pathways that exhibited shared significance. We specifically chose 5 visualizations: Focal adhesion, The ECM receptor interaction, and interactions involving immune cells and microRNAs in the tumor microenvironment (**Figure 4D**). These results suggest that the LOX family not only participates in tumor biological processes but may also influence the effectiveness of immunotherapy by modulating the immune microenvironment.

### An association study on the LOX family and immune cell infiltration using multidimensional correlation analysis

**Figure 5:**
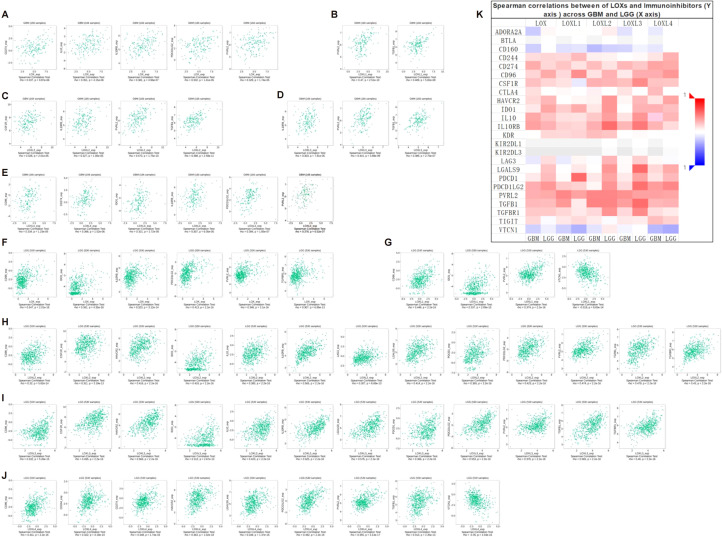
Correlation between LOX family expression and immune checkpoints in GBM and LGG. **(A)** Expression of LOXs positively correlates with CD274, IL10, IL10RB, PDCD1LG2, and PVRL2 in GBM. **(B)** Expression of LOXL1 positively correlates with PVRL2 and TGFB1 in GBM. In GBM, **(C)** expression of LOXL2 positively correlates with CSF1R, IL10RB, PVRL2, and TGFB1. **(D)** Expression of LOXL3 positively correlates with IL10RB, PVRL2, and TGFB1 in GBM. **(E)** In GBM, LOXL4 expression positively correlates with CD96, CD274, ID01, IL10RB, PDCD1LG2, and PVRL2. **(F)** Expression of LOX positively correlates with CD96, ID01, IL10RB, PDCD1LG2, PVRL2, and TGFBR1 in LGG. **(G)** In LGG, LOXL1 expression positively correlates with CD96, IDO1, PVRL2, and VTCN1. **(H)** In LGG, LOXL2 expression positively correlates with CD96, CSF1R, HAVCR2, IDO1, IL10, IL10RB, LAG3, LGALS9, PDCD1, PDCD1LG2, PVRL2, TGFB1, and TGFBR1. **(I)** In LGG, LOXL3 expression positively correlates with CD96, CSF1R, HAVCR2, ID01, IL10, IL10RB, LGALS9, PDCD1, PDCD1LG, PVRL2, TGFB1, and TGFBR1. **(J)** In LGG, LOXL4 expression positively correlates with CD96, CD244, CD274, HAVCR2, LGALS9, PDCD1LG2, PVRL2, TGFB1, and VTCN1. **(K)** Heatmap depicting the correlation between LOXs and immune checkpoints in GBM and LGG.

Additionally, we interrogated the TCIA database to investigate whether high expression of members from the LOX family confers benefits to immune checkpoint inhibitors. The results indicated that every member of the LOX family demonstrated an association with specific immunosuppressive agents in GBM (**Figures 5A-E**) and LGG (**Figures 5F-J**), thereby presenting evidence for the potential application of the LOX family in immunotherapy. For instance, CD96, IDO1, and PVRL2 display associations with all LOXs, whereas IL10RB and PDCD1LG2 are associated with all LOXs except LOX1. Subsequently, we conducted a correlation analysis between HAVCR2, LGALS9, and TGFB1 and LOXL2, LOXL3, and LOXL4. IL10 and TGFBR1 are associated with LOX, LOXL2, and LOXL3. CSF1R and PDCD1 are associated with LOXL2 and LOXL3. In addition, CD274 is associated with LOX and LOXL4. VTCN1 is associated with LOXL1 and LOXL4. Finally, the data above were integrated into a heat map, demonstrating the association between the LOX family and immunosuppressants in GBM and LGG (**Figure 5K**). These results reveal the complex role of the LOX family in regulating immune cell infiltration, supporting the hypothesis that it may promote tumor progression by affecting the immune microenvironment.

### The impact of the LOX family on immune cell infiltration in somatic cell copy number alterations

**Figure 6:**
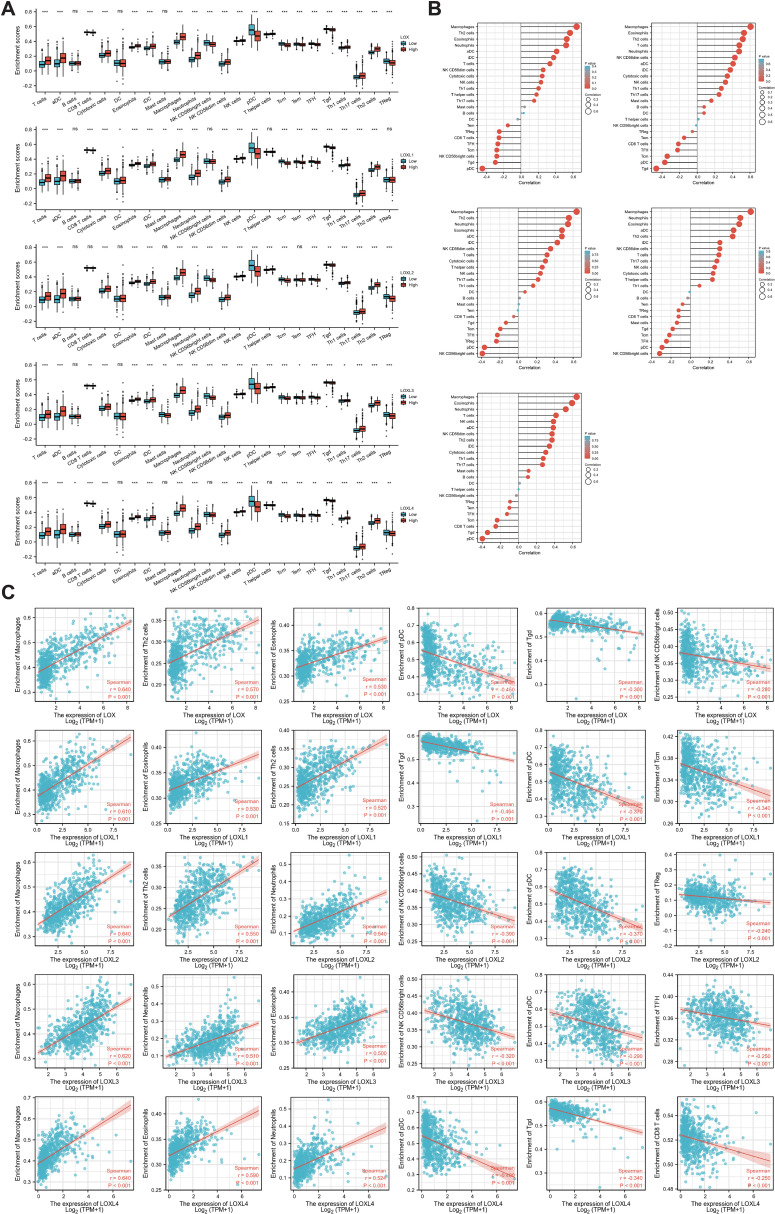
Correlation between immune infiltration levels and LOX family expression in glioma. **(A)** Spearman analysis of immune cell infiltration among 24 highly expressed glioma cell types. **(B)** Relationship between immune cell infiltration and LOX expression profiles. **(C)** Scatter plots show the top 6 immune cells most correlated with each LOX family member. *P < 0.05, **P < 0.01, ***P < 0.001; ns, not significant.

To investigate the presence of LOX family members and 24 different immune cell types in glioblastoma, we employed ssGSEA and Spearman correlation analysis. This analysis allowed us to demonstrate their infiltration into the glioblastoma microenvironment. The study findings revealed a positive correlation between the expression of LOX family members and immune cell infiltration, except B cells, dendritic cells (DCs), mast cells, NK CD56 bright cells, T helper cells (Th cells), regulatory T cells (Treg cells), and LO8T cells. Moreover, LOXL2 expression was observed in DCs, mast cells, and Tem cells, while LOXL3 showed expression in DCs. Additionally, LOXL4 did not exhibit expression in mast cells, NK CD56 bright cells, T helper cells, and dendritic cells, as depicted in **Figure 6A**.

**Figure 7:**
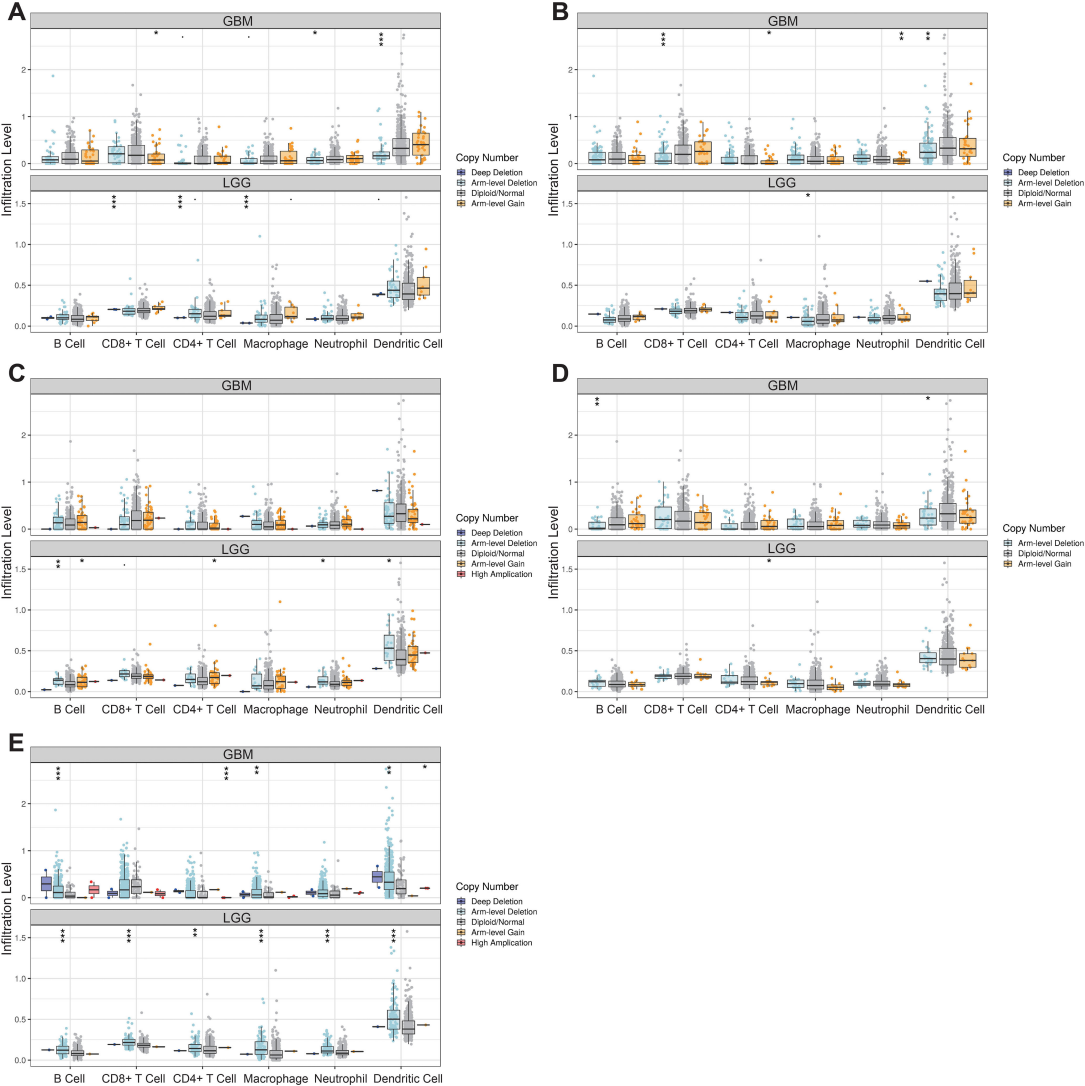
Correlation between changes in somatic copy number alterations and immune cell infiltration levels in glioma. *P < 0.05, **P < 0.01, ***P < 0.001.

Furthermore, we performed a Spearman correlation analysis (**Figure 6B**) to examine the correlation between the LOX family and immune cell infiltration. The results demonstrated a positive correlation between the expression of macrophages, eosinophils, neutrophils, Th2 cells, T cells, aDC, and iDC cells with LOX family members. Conversely, a negative correlation was found between pDC, TgdCD8T cells, and traditional Chinese medicine with LOX. We chose the six immune cells with the strongest correlation for each LOX family member to provide a more intuitive representation of this relationship. These were then visualized using a scatterplot, as shown in **Figure 6C**. Furthermore, additional evidence illustrates the correlation between somatic copy number alterations (SCNA) and the extent of immune cell infiltration, thereby offering novel insights into the involvement of the LOX family in the immune microenvironment (**Figure 7**).

### Expression of the LOX family in gliomas and its impact on cellular behavior

**Figure 8:**
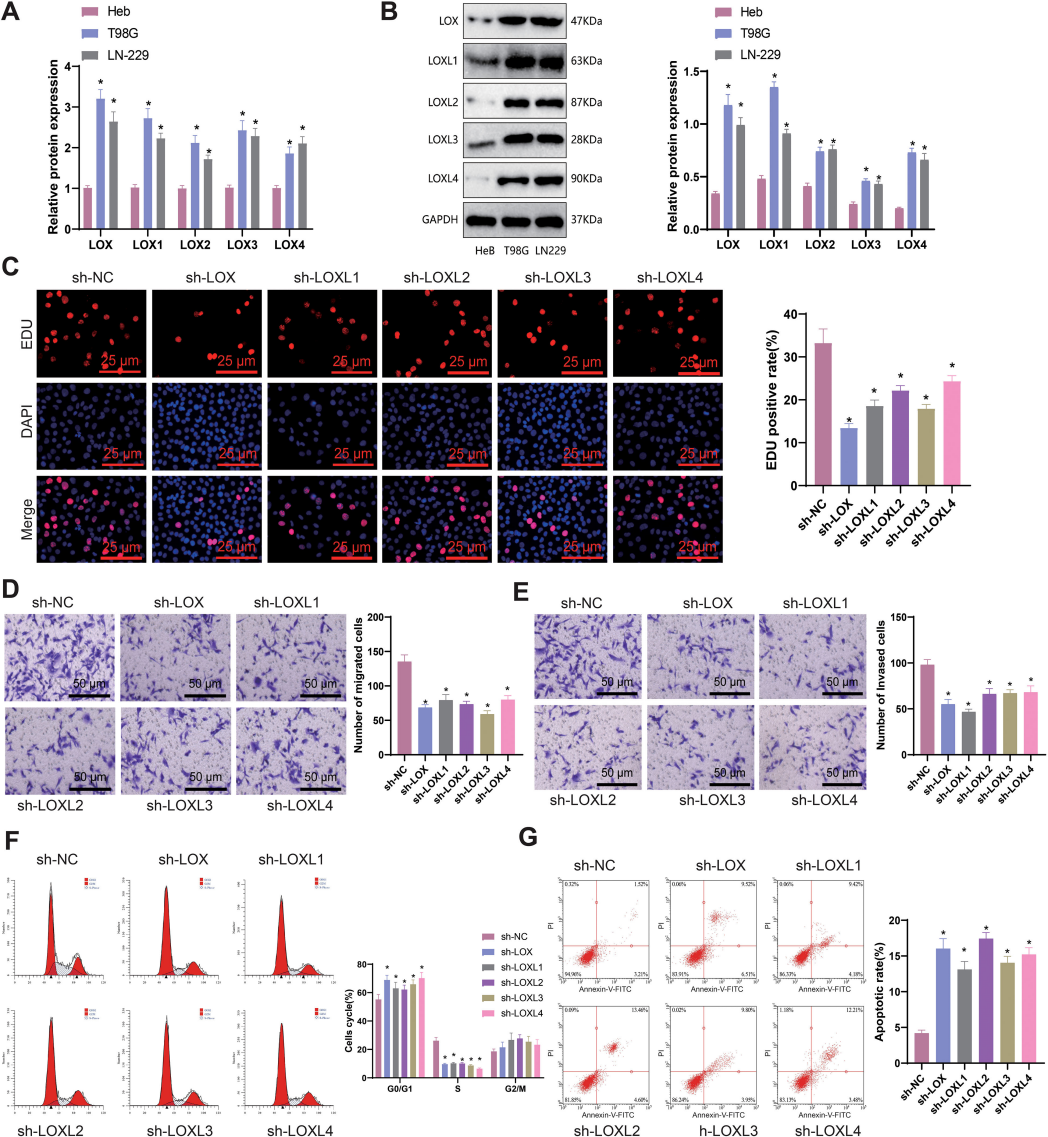
Effects of LOX family factors on proliferation, migration, and invasion of glioma cells. **(A)** mRNA expression of LOX, LOXL1, LOXL2, LOXL3, and LOXL4 in normal human glial cell line Heb and glioma cell lines T98G and LN-229 as detected by qRT-PCR. **(B)** Protein expression of LOX, LOXL1, LOXL2, LOXL3, and LOXL4 in normal human glial cell line Heb and glioma cell lines T98G and LN-229 as detected by WB. **(C)** Proliferation capacity of glioma cells assessed by EdU staining (200×). **(D)** Migration capacity of glioma cells assessed by Transwell assay (200×). **(E)** Invasion capacity of glioma cells assessed by Transwell assay (200×). **(F)** Cell cycle changes of glioma cells as analyzed by flow cytometry. **(G)** Apoptosis of glioma cells as determined by flow cytometry. * indicates a significant difference compared to the control group (sh-NC), p < 0.05. All cell experiments were replicated 3 times.

Our bioinformatics analysis revealed high expression of the LOX family in gliomas. To investigate the impact of the LOX family on glioblastoma, we analyzed the expression of LOX family members (LOX, LOXL1, LOXL2, LOXL3, and LOXL4) in normal human astrocytes (Heb) and glioblastoma cell lines (T98G and LN-229). The analysis was performed using qRT-PCR and Western blot techniques. The results indicated that members of the LOX family, including LOX, LOXL1, LOXL2, LOXL3, and LOXL4, exhibited high expression levels in glioma cells in comparison to normal human glial cell lines Heb as well as glioblastoma cell lines T98G and LN-229 (**Figures 8A, B**). Considering that LOX family factors are expressed at higher levels in T98G cells, we selected it as the subject for further experiments.

To investigate the influence of LOX family factors on the biology of gliomas, we utilized lentivirus-mediated silencing and validated the efficiency of silencing for each shRNA using qRT-PCR. The results indicate that different short hairpin RNAs (shRNAs) derived from the LOX family can effectively suppress the expression of their respective LOX family factors. Hence, we chose the shRNA exhibiting higher silencing efficiency for further experiments (**Supplementary Figure S1**).

EdU staining was conducted to assess the proliferation capacity of glioma cells, while the Transwell assay was used to evaluate their migration and invasion abilities. Flow cytometry was employed to examine the cell cycle and apoptosis rate. The results demonstrated that in comparison to the sh-NC group, the proliferation, migration, and invasion abilities of T98G cells were significantly reduced in the sh-LOX, sh-LOXL1, sh-LOXL2, sh-LOXL3, and sh-LOXL4 groups. Furthermore, there was a significant increase in the proportion of cells in the G1 phase, a notable decrease in the S phase, and a significant elevation in the cell apoptosis rate (**Figures 8C-G**).

The findings above suggest that inhibiting LOX family factors can effectively impede gliomas’ proliferation, migration, and invasion capabilities while also promoting cellular apoptosis.

### Silencing LOX family factors impacts the anti-tumor immune response of glioma cells, specifically the regulation of M2 macrophages and CD8^+^ T cells

**Figure 9:**
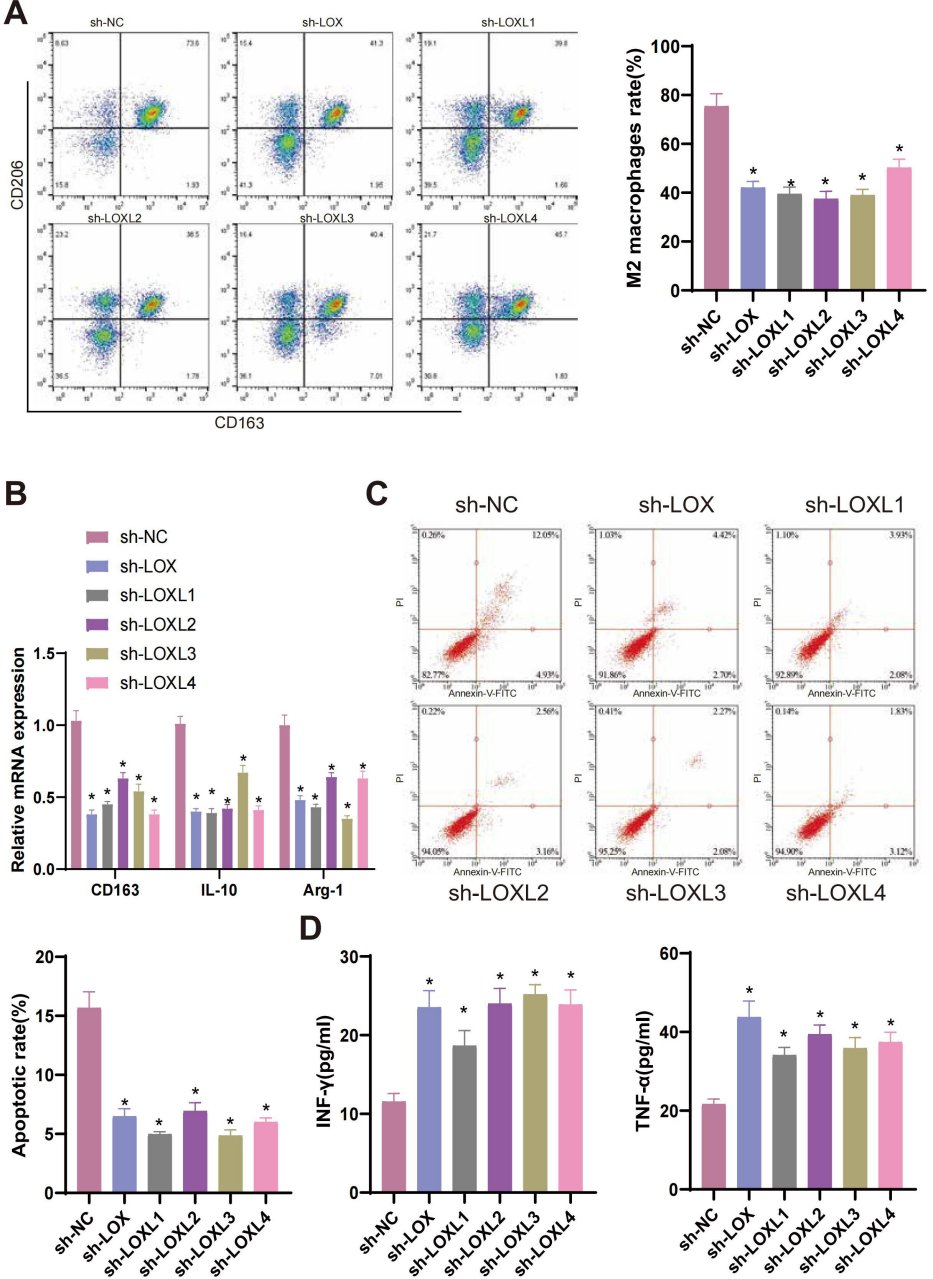
LOX family factors mediate anti-tumor immune responses in glioma cells. **(A)** Proportion of M2 macrophages induced by co-culture with T98G cells as determined by flow cytometry. **(B)** mRNA expression of CD163, IL-10, and Arg-1, markers of M2 macrophages, as detected by qRT-PCR. **(C)** Flow cytometry determined the Apoptosis of CD8+ T cells co-cultured with T98G cells. **(D)** Expression levels of IFN-γ and TNF-α in the co-culture medium of T98G cells and CD8^+^ T cells were measured by ELISA. * indicates a significant difference compared to the control group (sh-NC), p < 0.05. All cell experiments were replicated 3 times.

Previous studies have reported that M2 macrophages secrete transforming growth factor-β1 (TGF-β1) to enhance the migration and invasion of glioma cells (81). M2 macrophages generally facilitate the proliferation of cells, synthesis of the extracellular matrix, and remodeling of tissues, thereby contributing to the advancement of tumors (82, 83). To examine the influence of LOX family factors on macrophage proportion, we co-cultured T98G cells with LOX family factors silenced with THP-1 cells differentiated into macrophages. Flow cytometry determined the proportion of induced M2 macrophages in the co-cultured THP-1 cells. The results demonstrate a significant reduction in the proportion of M2 macrophages in T98G cells co-cultured with sh-LOX, sh-LOXL1, sh-LOXL2, sh-LOXL3, and sh-LOXL4 groups compared to the sh-NC group (**Figure 9A**). The expression of M2 macrophage markers CD163, IL-10, and Arg-1 mRNA in co-cultured THP-1 cells was determined using qRT-PCR. A significant decrease in the expression of CD163, IL-10, and Arg-1 mRNA, which are indicative of M2 macrophage polarization, was observed in THP-1 cells co-cultured with sh-LOX, sh-LOXL1, sh-LOXL2, sh-LOXL3, and sh-LOXL4 groups when compared to the sh-NC group (**Figure 9B**).

CD8^+^ T cells, which are cytotoxic, play a pivotal role in anti-tumor activity (84). Previous research has demonstrated that the infiltration of CD8^+^ T cells is a predictive factor for the prognosis of patients with glioblastoma (GBM). The inhibition of PTPN6 can potentially augment the infiltration of CD8^+^ T cells, enhancing the anti-tumor immune response and subsequently improving the clinical prognosis of GBM patients. Therefore, PTPN6 represents a promising immunotherapeutic target for treating GBM (85). To examine the effects of LOX family factors on CD8^+^ T cells, we conducted co-culture experiments in which T98G cells with silenced LOX family factors were co-cultured with CD8^+^ T cells. The apoptosis of CD8+ T cells was then assessed using flow cytometry. The apoptosis rate of CD8^+^ T cells co-cultured with the sh-LOX, sh-LOXL1, sh-LOXL2, sh-LOXL3, and sh-LOXL4 groups was significantly lower than that of the sh-NC group (**Figure 9C**). ELISA detection of IFN-γ and TNF-α expression in the culture medium revealed a significant increase in their levels compared to the sh-NC group. This increase was observed in the culture medium co-cultured with the sh-LOX, sh-LOXL1, sh-LOXL2, sh-LOXL3, and sh-LOXL4 groups (**Figure 9D**).

The results suggest that suppressing the Lox family factor can modulate the anti-tumor immune response in glioma cells by inhibiting M2 macrophage polarization and enhancing CD8^+^ T cell activity.

### The inhibitory effect of silencing LOX family factors on glioblastoma xenografts in mice and its impact on regulating immune cells

The *in vitro* cell experiments and bioinformatics analysis results confirm that silencing LOX family factors can inhibit gliomas’ proliferation, migration, and invasive abilities. Moreover, these factors mediate M2 macrophage polarization and enhance CD8^+^ T cell activity, thereby resulting in the immune suppression of glioblastoma cells. To determine whether this mechanism impacts tumor formation in mice, we injected glioma cells with silenced LOX family factors and established a glioma xenograft tumor model.

**Figure 10:**
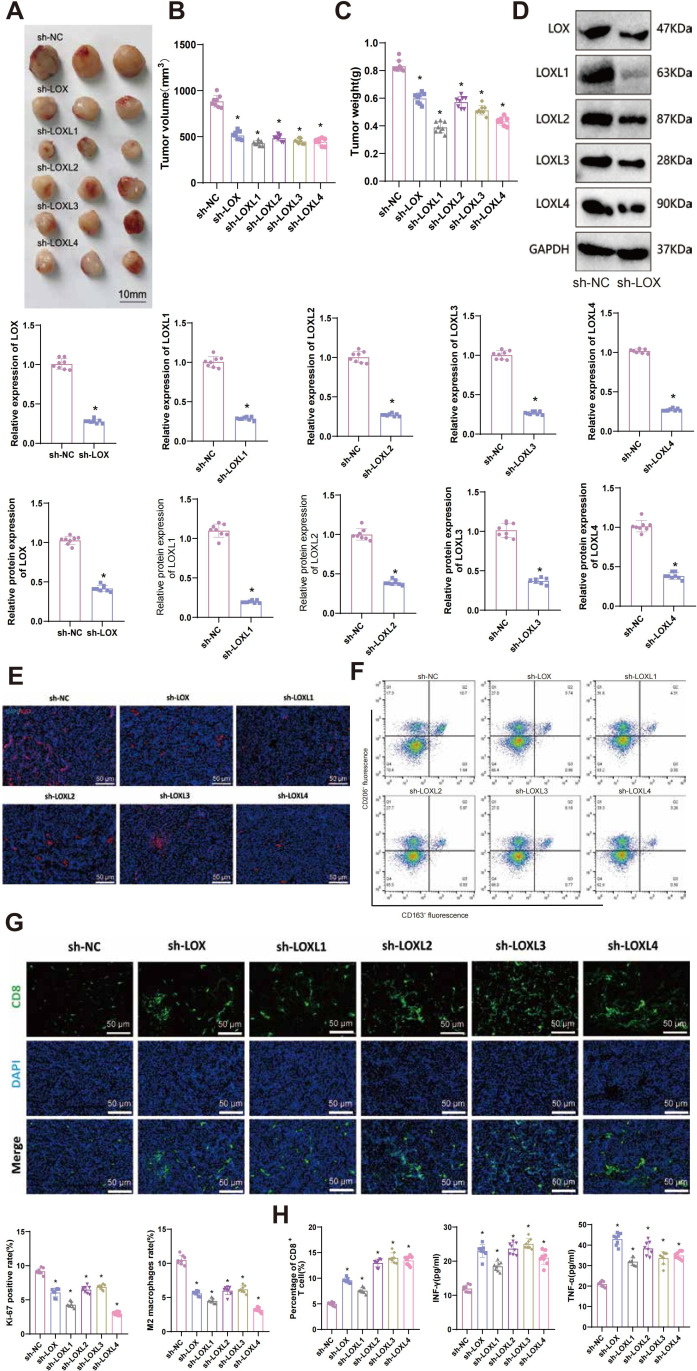
Effects of LOX family factors on tumorigenicity, immune cell infiltration, and exhaustion in glioma *in vivo*. **(A)** Morphology of tumor tissues from each group of mice. **(B)** Statistics of tumor volume in each group of mice. **(C)** Statistics of tumor weight in each group of mice. **(D)** Expression levels of LOX, LOXL1, LOXL2, LOXL3, and LOXL4 in tumor tissues as detected by RT-qPCR and WB. **(E)** Immunofluorescence staining of Ki67 protein expression in tumor tissues from each group of mice. **(F)** Proportion of M2 macrophages in tumor tissues from each group of mice as determined by flow cytometry. **(G)** Percentage of CD8^+^ T cells in tumor tissues from each group of mice as detected by immunofluorescence staining. **(H)** Expression levels of IFN-γ and TNF-α in tumor tissues from each group of mice were measured by ELISA. * indicates a significant difference compared to the control group (sh-NC), p < 0.05. N=8.

Tumor volume is regularly observed and measured in our study. Preliminary observations indicate a decrease in the tumor volume and weight of mice in the sh-LOX, sh-LOXL1, sh-LOXL2, sh-LOXL3, and sh-LOXL4 groups compared to the sh-NC group (**Figures 10A-C**).

RT-qPCR and WB techniques were used to detect the expression of LOX, LOXL1, LOXL2, LOXL3, and LOXL4 in tumor tissues. The results demonstrated a significant decrease in the mRNA and protein levels of the respective LOX family factors in the tumor tissues of each silencing group compared to the sh-NC group (**Figure 10D**).

The proliferation-related factor Ki67 protein level was detected using immunohistochemistry (IHC) in tumor tissues from each group. The results demonstrated a significant decrease in the expression of Ki67 protein in tumor tissues from sh-LOX, sh-LOXL1, sh-LOXL2, sh-LOXL3, and sh-LOXL4 mice, compared to the sh-NC group (**Figure 10E**). Flow cytometry analysis revealed a significant reduction in the proportion of M2 macrophages in the tumor tissues of sh-LOX, sh-LOXL1, sh-LOXL2, sh-LOXL3, and sh-LOXL4 groups of mice compared to the sh-NC group (**Figure 10F**).

Immunofluorescent staining revealed increased infiltration and exhaustion of CD8+ T cells in the tumor tissues of mice in the sh-LOX, sh-LOXL1, sh-LOXL2, sh-LOXL3, and sh-LOXL4 groups, compared to the sh-NC group (**Figure 10G**). The levels of immune-related factors INF-γ and TNF-α in mouse tumor tissues were assessed using ELISA. The results indicated a significant increase in the expression of INF-γ and TNF-α in the tumor tissues of mice from the sh-LOX, sh-LOXL1, sh-LOXL2, sh-LOXL3, and sh-LOXL4 groups compared to the sh-NC group (**Figure 10H**).

These findings imply that suppressing LOX family factors could impede the tumorigenicity of gliomas *in vivo* and influence the infiltration and exhaustion of immune cells. Consequently, it could inhibit M2 macrophage polarization while enhancing the activity of CD8^+^ T cells. Further validation confirmed the key role of the LOX family in gliomas and its ability to regulate the immune microenvironment.

## Discussion

Glioma, a prevalent tumor in the central nervous system, has consistently remained a prominent subject in medical research, focusing on its treatment and prognosis (5, 6). Recently, the LOX family has gained significant attention due to its vital role in multiple types of cancer (86–89). This study aims to comprehensively investigate the expression patterns and functions of the LOX family in gliomas and examine their association with clinical characteristics.

Our research revealed a significant increase in the expression of the LOX family in gliomas, with a pattern of expression closely linked to patient prognosis. These findings offer a fresh perspective on the molecular classification of gliomas and have the potential to assist in future clinical decision-making, particularly in selecting more precise treatment options (6, 90, 91).

We have discovered the interactions between the LOX family and numerous genes and proteins using high-throughput sequencing and bioinformatics analysis. These interactions potentially affect tumor development, invasion, and migration. These findings provide valuable insights for uncovering the molecular mechanisms underlying glioblastoma (92, 93). By conducting a comprehensive analysis of the biological functions of the LOX family in glioblastoma, we discovered that it potentially influences the development of this disease by promoting tumor cell proliferation, migration, and invasion while inhibiting cell apoptosis. Furthermore, the LOXs family can potentially modify the biological behavior of gliomas by impacting the tumor microenvironment and immune response. These findings establish a theoretical foundation for developing therapeutic strategies that target the LOX family.

This study revealed a complex interaction between the LOXs family and immune infiltration. Our findings indicate a positive correlation between the expression of the LOXs family and the infiltration of macrophages and eosinophils, whereas a negative correlation was observed with the infiltration of Treg and CD8^+^ T cells. These findings indicate that the LOX family might impact the immune microenvironment of glioblastoma by regulating immune cell infiltration and activity (94, 95). Specifically, the family of LOX can generate an immunosuppressive microenvironment within a tumor by promoting the polarization of M2 macrophages and suppressing the activity of CD8^+^ T cells. This finding offers a fresh perspective for comprehending the mechanisms by which gliomas obtain immune evasion and advancing immunotherapy strategies.

Although functional experiments in this study indicate that LOX gene knockdown can affect the polarization of M2 macrophages and the activity of CD8+ T cells, the specific molecular mechanisms remain unclear. LOX family members may affect the tumor immune microenvironment through the JAK/STAT signaling pathway. In colon cancer, CXCL8 promotes M2 macrophage polarization by activating the STAT3 signaling pathway and upregulating PD-L1 expression in M2 macrophages while simultaneously reducing T cell infiltration (96). Additionally, studies have found that drugs can effectively reverse M2-to-M1 polarization via the co-delivery of STAT6 inhibitors, suppressing tumor growth and metastasis (97). Future experiments could verify this by silencing LOX family members and assessing M2 macrophage polarization markers, CD8+ T cell infiltration, and the expression levels of STAT3 signaling pathway proteins.

Considering the significant expression and diverse functions exhibited by the LOX family in gliomas, asserting that they could serve as valuable therapeutic targets is plausible. Future research can examine the therapeutic effects of small molecule inhibitors or antibodies that target the LOX family (98, 99). Our research indicates that therapeutic approaches focused on the LOXs family can potentially enhance treatment effectiveness for glioblastoma by suppressing tumor cell malignancy and modifying the tumor immune microenvironment. Moreover, inhibiting the LOXs family can improve the effectiveness of other therapeutic interventions like radiotherapy and chemotherapy. Nevertheless, developing therapeutic drugs targeting the LOXs family encounters several challenges, such as drug selectivity, specificity, and toxicity. These challenges necessitate further research.

**Figure 11:**
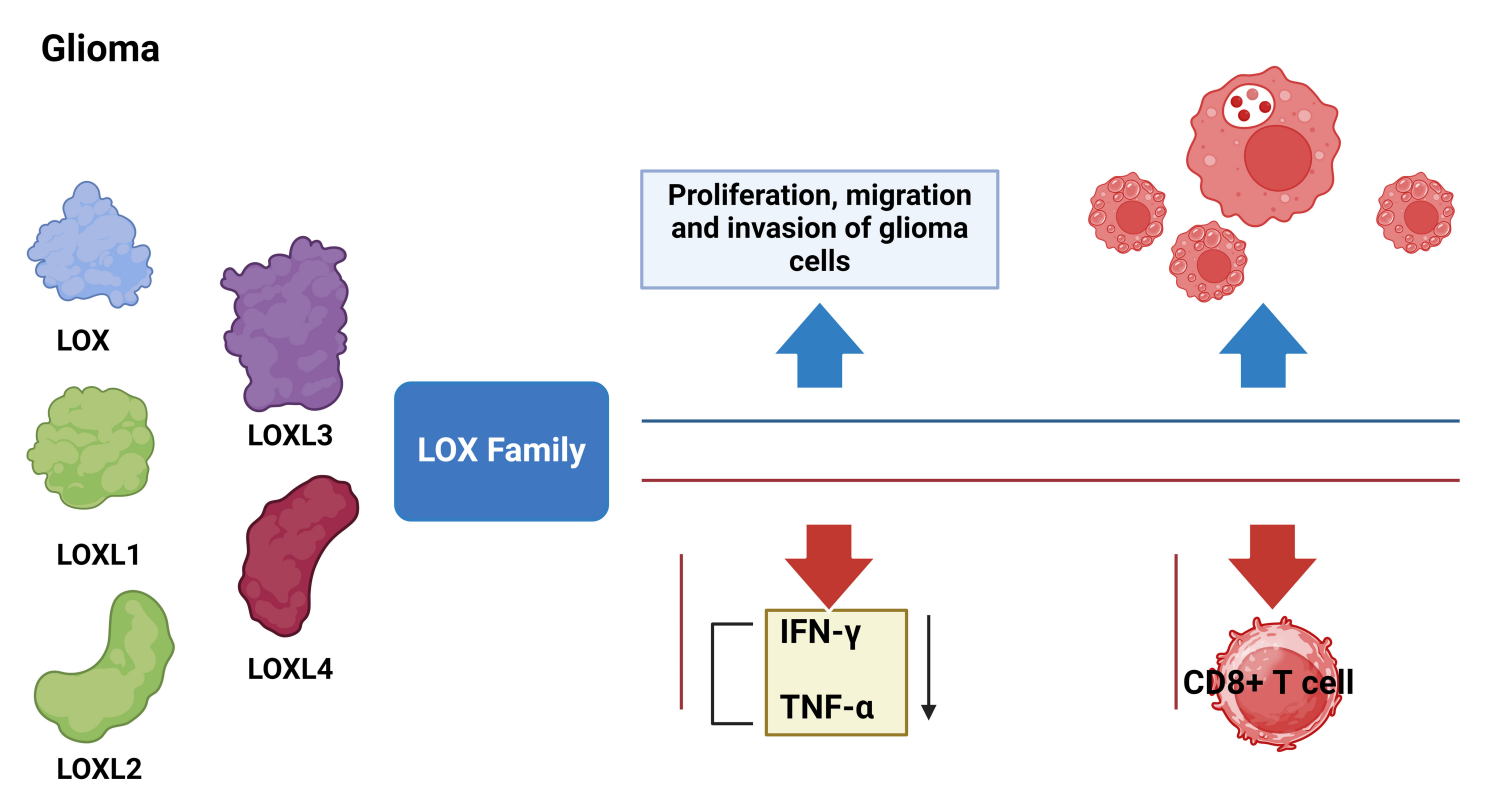
Molecular mechanisms of LOX family genes in the occurrence and development of glioma. Blue arrows indicate LOX activation and red arrows indicate LOX silencing.

The interaction between the LOXs family and immune cells offers potential for its application in immune therapy. Our research revealed that suppressing the LOXs family can modify the tumor’s immune microenvironment and augment the infiltration and activity of immune cells, consequently enhancing the effectiveness of immunotherapy. This discovery offers novel strategies and targets for the immunotherapy of gliomas. However, additional research and exploration are still required to determine effective strategies for combining the LOXs family with existing immunotherapy methods. Additionally, efforts should focus on optimizing treatment plans to maximize therapeutic effects. In summary, we draw the following conclusions: the high expression of the LOXs family in gliomas is related to poor prognosis, level of immune infiltration, and responsiveness to immune suppressants. Therefore, the LOXs family can serve as an important prognostic biomarker for gliomas and a potential therapeutic target and sensitizer for immunotherapy (**Figure 11**).

This study has several limitations. First, the small sample size may impact the reliability and representativeness of the results. Second, the study is based on laboratory data without validation in clinical samples, limiting the clinical applicability (100, 101). Additionally, the mechanisms of the LOX family in gliomas, including interactions with HIF-1α and TGF-β signaling pathways, require further exploration. The potential toxicity and selectivity of LOX inhibitors in clinical treatment were not fully assessed, which should be addressed in future studies. Although we hypothesize that the LOX family could sensitize immunotherapy, its synergy with immune checkpoint inhibitors has not been tested in clinical samples. Future research should validate these findings in larger clinical cohorts and explore the specific mechanisms of the LOX family in gliomas. Furthermore, while we conducted a stratified analysis of GBM and LGG, we did not further subdivide LGG by WHO grade II vs. III or IDH status (IDH-mutant vs. IDH-wildtype) due to data limitations, which may influence LOX expression. Larger datasets are needed to confirm these potential differences.

Based on the findings of this study, it is recommended that future research should delve deeper into understanding the biological functions and mechanisms of the LOXs family in gliomas, along with their specific interactions with immune cells. Furthermore, developing and optimizing therapeutic strategies targeting the LOXs family is imperative. Additionally, investigating the potential of integrating these strategies with other treatment methods is crucial for future research. Through comprehensive research and unwavering dedication, we believe that the LOXs family will contribute to revolutionary breakthroughs in treating gliomas.

In summary, this study has revealed the significant role and multiple biological functions of the LOXs family in glioblastoma, thereby offering new biomarkers and treatment targets for diagnosing and treating glioblastoma. The LOXs family is not only an important biomarker for predicting outcomes in glioblastoma, but it also has the potential to be a therapeutic target and a sensitizing agent for immunotherapy. Nevertheless, this study has limitations, including a small sample size and the absence of clinical validation, which should be addressed in future research. We aim to advance the clinical utilization of the LOXs family for treating glioma by conducting extensive research. It will offer additional hope and possibilities for patients with glioma.

## Data Availability

The original contributions presented in the study are included in the article/**Supplementary Material**. Further inquiries can be directed to the corresponding author.

## Supplementary material

Supplementary Figure 1 Silencing efficiency of each shRNA as detected by qRT-PCR. * indicates a significant difference compared to the control group (sh-NC), p < 0.05. All cell experiments were replicated 3 times.
