# Supplementary figures and images for: Exploring the role of LOX family in glioma progression and immune modulation
Source: Front Immunol. 2025 Apr 9;16:1512186. doi: 10.3389/fimmu.2025.1512186 (PMC12014642; doi:10.3389/fimmu.2025.1512186)

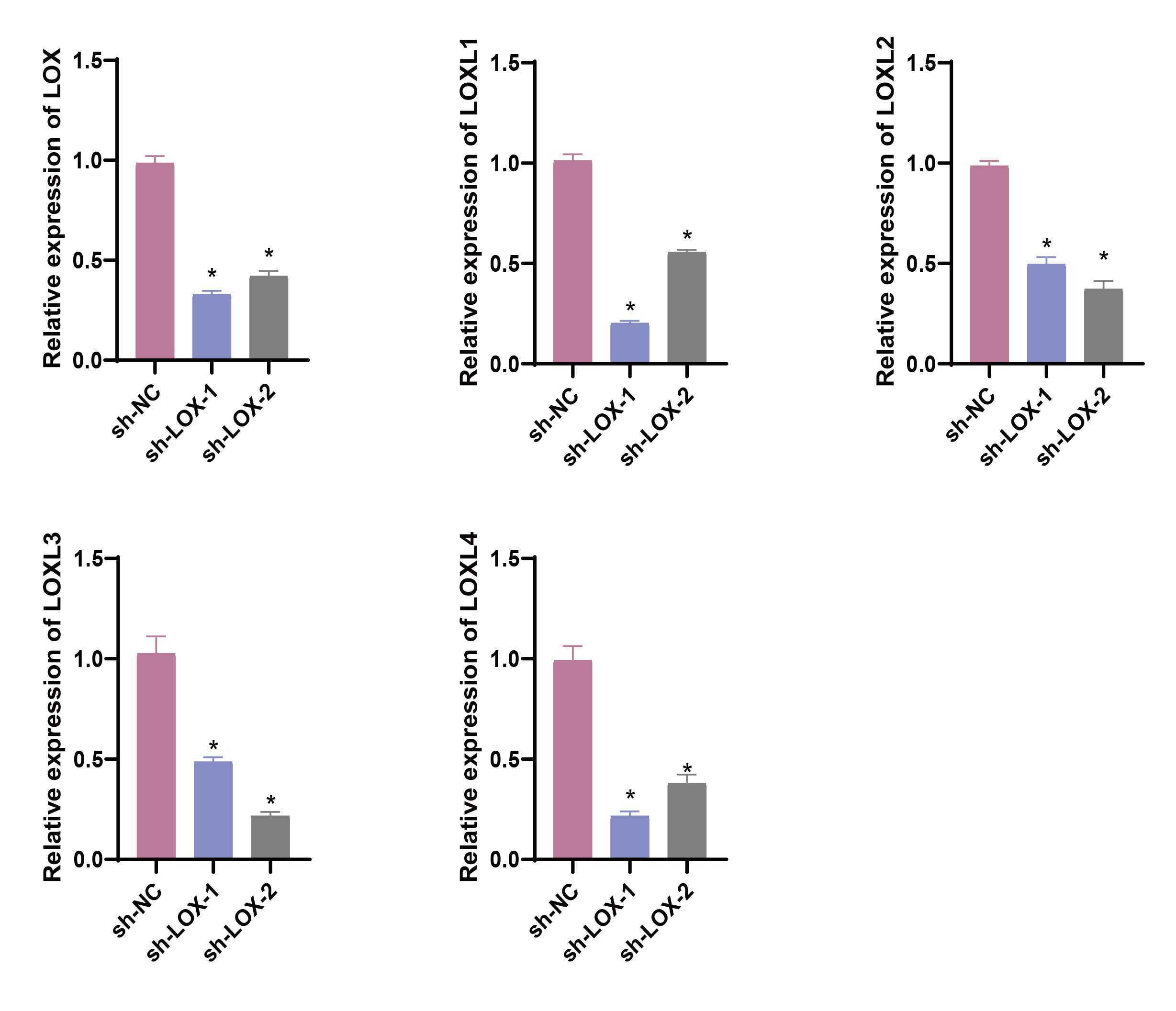

Supplement: Supplementary Figure 1 — Silencing efficiency of each shRNA as detected by qRT-PCR. * indicates a significant difference compared to the control group (sh-NC), p < 0.05. All cell experiments were replicated 3 times. [file Image1.jpeg]
