# Supplementary material for: Exploring the role of LOX family in glioma progression and immune modulation
Source: Front Immunol. 2025 Apr 9;16:1512186. doi: 10.3389/fimmu.2025.1512186 (PMC12014642; doi:10.3389/fimmu.2025.1512186)
Supplement: Supplementary file 2 [file Table1.docx]

**Table S1. shRNA Sequences**

| shRNAs | Sequence |
| --- | --- |
| sh-NC | 5’-TGGTTTACATGTCGACTAA-3’ |
| sh-LOX-1 | 5’-CTGCACAATTTCACCGTATTA-3’ |
| sh-LOX-2 | 5’-GCTGCACAATTTCACCGTATT-3’ |
| sh-LOXL1-1 | 5’-ACGTGGTGAGATGCAACATTC-3’ |
| sh-LOXL1-2 | 5’-CACGTGAACCCAAAGTATATT-3’ |
| sh-LOXL2-1 | 5’-GAAACCCTCCAGTCTATTATA-3’ |
| sh-LOXL2-2 | 5’-GGCAATGAGAAGTCCATTATA-3’ |
| sh-LOXL3-1 | 5’-ACTGGGACTCTGGGAATATAA-3’ |
| sh-LOXL3-2 | 5’-GGACCCACAGTGCCAAATATG-3’ |
| sh-LOXL4-1 | 5’-TTGGACCAGTGCGGGTCTAAT-3’ |
| sh-LOXL4-2 | 5’-CCTGACGAATAAGAACTCCTT-3’ |

**Table S2. RT-qPCR primer sequences**

| Gene | Sequence |
| --- | --- |
| LOX (human) | F 5-AGGGTGAGGAGTAAGGGACC -3  R 5-GGAAATCTGAGCAGCACCCT-3 |
| LOXL1 (human) | F 5-TGTACCGGCCCAACCAGAA-3  R 5-CCGGTTGGGGAGGAAGTCTG -3 |
| LOXL2 (human) | F 5-GTGAGCTCAGACTTGGTGCT-3  R 5-CAAGGGCCCAGATGTCCAAT-3 |
| LOXL3 (human) | F 5-AATGTCATTGAGGCCCGTGT-3  R 5- GCGAACTTCACTCAGGTGGA-3 |
| LOXL4 (human) | F 5- CGCGCTCCATCTGGTATCTT-3  R 5- GTTGTCATCACACACGGTGC-3 |
| LOX (mouse) | F 5- CAGAACGGCTTGTGTAACTGC-3  R 5- TGCCCGTTGTTCTCCCATTG-3 |
| LOXL1 ( mouse ) | F 5-ATTGCCCGGGTGCTGAATAA -3  R 5-ACACAAAGGCTGAAACCCCA -3 |
| LOXL2 ( mouse ) | F 5-GGCGCTTCCAGACAGAGTTC -3  R 5-CGGACCTGGATCTTGACCAC -3 |
| LOXL3 ( mouse ) | F 5-GGATGCTGAAAGGGCACAGA -3  R 5-GCTTGCCGTTTCTTCGGAAC-3 |
| LOXL4 ( mouse ) | F 5-ATTCACCGGGTTGACTGTCTG -3  R 5-GCACTTTCAGCTCCTGCTCTG -3 |
| GAPDH (mouse) | F 5- CCCTTAAGAGGGATGCTGCC-3  R 5-TACGGCCAAATCCGTTCACA -3 |
| CD163 (human) | F 5- TCCTTGTGGGATTGTCCTGC -3  R 5- TGGGAATTTTCTGCAAGCCG -3 |
| IL-10 (human) | F 5- GGGCACCCAGTCTGAGAAC -3  R 5- TCACATGCGCCTTGATGTCT -3 |
| Arg-1 (human) | F 5- ACTTAAAGAACAAGAGTGTGATGTG -3  R 5- CATGGCCAGAGATGCTTCCA -3 |
| GAPDH (human) | F 5-GACAGTCAGCCGCATCTTCT-3  R 5-GCGCCCAATACGACCAAATC-3 |

**Table S3. Details of the first antibody product**

| Name | Cat. | Dilution ratio | Manufacturer | Country | MW (kDa) |
| --- | --- | --- | --- | --- | --- |
| LOX | ab174316 | 1: 1000 | Abcam | UK | 47 |
| LOXL1 | ab313585 | 1: 1000 | Abcam | UK | 63 |
| LOXL2 | ab96233 | 1: 1000 | Abcam | UK | 87 |
| LOXL3 | ab232878 | 1: 1000 | Abcam | UK | 28 |
| LOXL4 | ab313797 | 1: 1000 | Abcam | UK | 90 |
| GAPDH | ab9485 | 1: 2000 | Abcam | UK | 37 |
